# We Need to Talk About Complexity in Health Research: Findings From a Focused Ethnography

**DOI:** 10.1177/1049732320968779

**Published:** 2020-11-06

**Authors:** Chrysanthi Papoutsi, James Shaw, Sara Paparini, Sara Shaw

**Affiliations:** 1 University of Oxford, Oxford, United Kingdom; 2 Women’s College Hospital, Toronto, Ontario, Canada; 3 University of Toronto, Toronto, Ontario, Canada

**Keywords:** complexity, applied health research, boundaries, qualitative research, focused ethnography, UK

## Abstract

There is increasing focus on complexity-informed approaches across health disciplines. This attention takes several forms, but commonly involves framing research topics as “complex” to justify use of particular methods (e.g., qualitative). Little emphasis is placed on how divergent and convergent ways of knowing complexity become negotiated within academic communities. Drawing on findings from a focused ethnography of an international workshop, we illustrate how health researchers employ “boundary-ordering devices” to navigate different meanings ascribed to complexity while they attempt to sustain interdisciplinary communication and collaboration. These include (a) surfacing (but not resolving) tensions between philosophical grounding of knowledge claims and need for practical purchase, (b) employing techniques of representation and abstraction, and (c) drawing on the fluid, ongoing accomplishment of complexity for different audiences and purposes. Our findings have implications for progressing complexity-informed health research, particularly with respect to qualitative approaches.

## Introduction

Complexity has become increasingly popular as a theoretical lens in applied health research over the past 30 years (Anderson et al., 2005; Greenhalgh & Papoutsi, 2018; Plsek & Greenhalgh, 2001). Widespread use has led to multiple interpretations and applications of complexity-informed approaches, including operational definitions (Braithwaite et al., 2018; Churruca et al., 2019; Rickles et al., 2007). Less attention has been paid to how complexity is negotiated across disciplinary traditions in applied health research and the role this plays in the development of the field. In this focused ethnography, we examine how health researchers leverage particular discourses to negotiate and justify the relevance of a complexity lens in their work, thereby structuring how interpretations of complexity become prioritized in applied health research. We ask, first, how do actors in applied health research navigate the philosophical assumptions that characterize their different disciplinary approaches to understanding complexity, while communicating about complexity across disciplinary boundaries? Second, how do concerns for the practical import of complexity influence the meanings ascribed to this concept? In so doing, we contribute to understanding how researchers engage with complexity across epistemic communities (academic and non-academic) and indicate ways in which complexity can be mobilized to extend interdisciplinary dialogue.

By applied health research, we refer to the intersecting disciplinary perspectives and methods examining practical efforts to improve public health, health care, and well-being more generally. Our intended audience is primarily qualitative health researchers seeking to draw on complexity in their work within applied health research, but findings have broader relevance for those drawing on other methodologies. Our article proceeds as follows. We first summarize select literature pertaining to complexity in applied health research to illustrate the context in which our study takes place. We then outline the theoretical framework that underlies our approach, describing the concept of *boundary-ordering devices* as it relates to the use of scientific concepts. The “Findings” section outlines three boundary-ordering devices mobilized by applied health researchers at an interdisciplinary workshop on complexity held at the University of Oxford in 2017. We conclude with a discussion on the ways complexity-informed health research becomes constituted and outline implications for qualitative health researchers interested in adopting a complexity perspective.

## A Brief Background on Complexity

The concept of complexity has a rich cross-disciplinary history (Gell-Mann, 1994; Hawe, 2015; Holland, 1992). The original roots of complexity theory include (a) Cybernetics, that is, the study of how elements of systems communicate and bring about order, synthesizing perspectives primarily from mathematics, engineering, and neurobiology, as well as anthropology and social psychology; (b) General Systems Theory, that is, the study of systems, taking the broad stance that many naturally occurring systems may be fruitfully understood as organic wholes that are inherently “open” (or are connected to systems that are inherently open); and (c) System Dynamics, that is, a mathematical approach to understanding the non-linear behavior of elements of naturally occurring systems (Abraham, 2011).

Intersections across these three fields have led to a collection of principles for the study of systems across physical, biological, and social sciences (Holland, 1992; Thrift, 1999). This meeting of ideas characterizes complexity theory, resulting in a cross-fertilized field of theoretical development and empirical applications in different substantive domains (Manson, 2001). The interdisciplinary history of complexity theory poses challenges for those seeking to articulate common underpinning principles of the concept (Thrift, 1999). Social scientists have proposed characteristics such as non-linear relationships, causation that is contingent on unpredictable influences, and the importance of focusing on emergent properties (Byrne, 2001); the potential of pragmatism as an epistemic foundation to complexity research (Long et al., 2018); with the centrality of these topics to complexity long debated across fields (Goldstein, 1999).

Mol and Law’s (2002b) edited collection on complexity was introduced as a counter to simplification in theorizing about science and technology. In their view, complexity exists when “things relate but don’t add up, if events occur but not within the processes of linear time, and if phenomena share a space but cannot be mapped in terms of a single set of three-dimensional coordinates” (Mol & Law, 2002a, p. 1). A complexity lens becomes relevant when linear ways of thinking fail to account for the multiple overlapping and divergent ways in which the world works. This observation conveys important insights for the study of complexity in applied domains; we need to find ways to draw attention to messiness without attempting to pin complexity down with any degree of specificity.

### Complexity in Applied Health Research

Recognition of multiple interacting influences on health-related interventions have motivated substantial attention to complexity in applied health research. In 2000, this culminated in the United Kingdom’s Medical Research Council (MRC) releasing a report that popularized the concepts of complexity and complex health interventions (Campbell et al., 2000). The primary motive behind what was to become known as “the MRC framework” was to provide a rigorous approach to developing and evaluating complex interventions beyond traditional randomized controlled trials. The framework focused on modifications to trial design that would enable stronger consideration of the many interacting components of complex interventions, using the development and implementation of a specialized hospital stroke treatment unit as an example.

The framework was updated in 2008 to reflect the insights and experiences that had accrued since its original publication (Craig et al., 2008). One of the primary motives for updating the framework was to incorporate “greater use of the insights provided by the theory of complex adaptive systems” (p. 1). In this spirit, the definition of a complex intervention was expanded from an intervention with multiple components that may act both independently and inter-dependently (Campbell et al., 2000), to one based on a more fulsome description of intervention characteristics, including the number and difficulty of behaviors required, the number of groups or organizational levels involved, the number and variability of outcomes and the degree of flexibility permitted (see Craig et al., 2008, for a detailed description). Further installments of the MRC framework have advocated for more detailed process evaluation to shed insight on implementation challenges (Moore et al., 2015), and different evaluation designs to better represent complexity (Lamont et al., 2016). Many of these attempts to articulate complexity center on specific assumptions about causal relationships among component parts of interventions, and about their emergent properties as interventions become implemented.

The publication and ongoing refinements to the MRC framework have invited much debate on the nature of complexity and best ways to address it in applied health research. A number of researchers have criticized the framework (Broer et al., 2017; Paley & Eva, 2011; Shiell et al., 2008), suggesting that it provides a simplistic version of complexity that conforms to, rather than challenges, the assumptions underlying overly simplified approaches to evaluation in health care and public health (Clark, 2013; Cohn et al., 2013). Critiques and theoretical assessments of MRC-inspired applications of complexity theory have focused on how complexity should be understood as a characteristic of interventions and/or of the systems into which they are introduced (Clark, 2013; Cohn et al., 2013; Shiell et al., 2008). A recent special issue sought a paradigm shift in complexity studies, from conventional research designs predicated on linearity and predictability toward theoretically grounded, methodologically pluralistic, flexible, and adaptive approaches (Greenhalgh & Papoutsi, 2018). These arguments represent a contested history of the concept of complexity in applied health research and helped to form the backdrop to the workshop on which our fieldwork focused.

Complexity studies have not just been carried out in the U.K. context as examples abound in other countries such as Australia (e.g., Braithwaite et al., 2017), Canada (e.g., Grudniewicz et al., 2018), the United States (e.g., Lanham et al., 2013), and Africa (Karemere et al., 2015; Mash et al., 2008). It is beyond the scope of this article to provide a full history of complexity theory in applied health and care. Pointers to other sources in applied health care are included in the BMC Medicine “Understanding Complexity in Health Systems: International Perspectives” special issue (https://www.biomedcentral.com/collections/complexity) and across disciplines in the 2018 Map of the Complexity Sciences by Brian Castellani (https://www.art-sciencefactory.com/complexity-map_feb09.html).

## Theoretical Lens

Our analysis is informed by theoretical work on boundary objects, focusing specifically on how they are constituted and maintained in interdisciplinary collaboration (Bowker et al., 2016; Star, 1999, 2010; Star & Griesemer, 1989). Susan Leigh Star described boundary objects as either material or abstract objects (including concepts) that are flexible enough to meet the practical needs of a diverse group of actors while “robust” enough to maintain some semblance of persistent identity (Bowker et al., 2016; Star & Griesemer, 1989). She emphasized that boundary objects “are a sort of arrangement that allows different groups to work together without consensus,” and the concept was established in part through the study of scientific collaboration (Bowker et al., 2016). Boundary objects “reside between social worlds” and have an identity that transcends a given community of practice, along with a more nuanced identity that characterizes their distinct use within specific communities (Star, 2010).

To examine engagement with complexity across epistemic communities, we drew parallels with early work by Shackley and Wynne (1996) who studied cooperation between science and policy in climate change. The authors suggest that, to talk about scientific uncertainty without challenging the authority of scientific knowledge or its potential for policy impact, environmental scientists formulated a number of discursive “boundary-ordering devices” (Shackley & Wynne, 1996). These devices were embedded in translational processes “for achieving some understanding among actors involved in highly fluid institutional and epistemic sets of relations” that allowed them “to define their interests, build alliances, map out futures, and construct identities rapidly and across many domains” (p. 280). For example, to achieve policy consensus with scientific claims, scientists had to clearly articulate ways of *auditing* uncertainty (as a boundary-ordering device) and making it visible (achieving “certainty about uncertainty” even though within the scientific community there was little agreement on how to do this). Similarly, scientists had to be seen as assuming the responsibility for *reducing* uncertainty through future research (as another boundary-ordering device), to sustain policy interest (and deflect responsibility from the policy process). Boundary-ordering devices become crucial in reconciling uncertainty and authority, primarily because they allow for heterogeneous interpretations to co-exist while enabling cooperation. Our study of boundary-ordering devices is similarly motivated by the effort to better understand how complexity is represented and mobilized in applied health research even when what counts as complex appears to have different meanings and purposes for different actors.

## Method

We conducted a focused ethnography of a 2-day, international workshop on complexity using a combination of observations and informal interviews (during the event) and document analysis (before and after the event). Focused ethnography typically involves short-term field visits, one or more researchers with “insider” knowledge, and intensive data collection (Knoblauch, 2005; Wall, 2014). Although it enables depth of insight as researchers remain focused on specific research questions, this methodological approach has been criticized for inconsistencies in its paradigmatic orientation and for allowing limited scope for immersion compared with conventional ethnographic approaches (Knoblauch, 2005; Wall, 2014). We have used focused ethnography as a way to indicate the “intent” and intellectual curiosity with which we approached interpretive data collection and analysis for this study, albeit in a relatively bounded fashion focused on the “situative performance of social actions” (Knoblauch, 2005). This approach made sense given our own background knowledge of complexity studies (some of the authors were involved in setting up the workshop), the short-term nature of organizing the event and our participant observation (6 months in total, including submission and review of abstracts), and the specific nature of our research questions. For other examples of focused ethnographies, see Cruz and Higginbottom (2013) and Wall (2013).

Ethics approval was granted by the Central University Research Ethics Committee at the University of Oxford (reference: R51769/RE001). We distributed participant information sheets to workshop attendees at registration, informing them that researchers would be collecting field notes for the duration of the workshop. Participants were invited to opt out from the study if they did not wish their contributions to be recorded, either by filling in an opt-out form or by letting the researchers know. No one opted out from the study at registration or during the workshop.

### Setting

The international “We Need to Talk About Complexity” workshop was hosted and sponsored by Green Templeton College at the University of Oxford in June 2017. The workshop brought together scholars from a range of disciplines within and beyond health care with the aim of opening up a space where the foundation of complexity would be debated. It was motivated in part by the increasing use of complexity theory to define and evaluate interventions in health care, and sought to identify missed opportunities for cross-disciplinary dialogue. Participants belonged to different research communities, including primary care and health services research, public health, sociology, social policy, and business and management. The open call for abstracts attracted high-quality submissions, of which 33 were accepted as oral or poster presentations.

The program was designed to maximize opportunities for active participation and cross-disciplinary engagement, with chairing deliberately encouraging exchanges across participants, disciplines, and viewpoints. It involved two short days (1–5 p.m. and 9–1 p.m.) with an evening reception and dinner on Day 1 and poster presentations (17 in total) during extended breaks. Following two keynote talks, paper sessions were organized around four topics:

1. Systems approaches to public health
2. Implementing and evaluating complex interventions—Time to revise language and theory?
3. Simulation, modeling, and data
4. Relational approaches, communication, and user-centered design

Sixty participants from eight countries attended (the United Kingdom, the United States, Canada, New Zealand, Australia, Belgium, Netherlands, and Spain). Presentations covered theoretical and philosophical debates, policy analysis, and applied research methodologies leveraging complexity theory as a conceptual approach. Dialogue ranged from addressing a lack of theoretical clarity, to debate regarding paradigmatic positioning, implications for methodological design, and relevance for policy-level decision-making.

### Data Collection

Data collection focused primarily on the workshop: the presentations, actors, interactions, and debate. We collected data through observation and informal discussions. Two authors acted as participant observers, taking contemporaneous field notes throughout the workshop (including capturing verbatim interaction). Where certain actors, presentations, or discussions were of particular interest, we followed up during breaks and/or in the evening and recorded notes as soon as possible thereafter.

We were interested in the wider debates around complexity theory in applied health research, the evolution of the workshop, and the interaction among the actors involved. Therefore, our research data included the original description of the workshop and call for abstracts, submitted abstracts and reviews, all (oral and poster) presentations, and a blog post summarizing the workshop after its completion. Finally, we included key texts (e.g., policy papers) that participants repeatedly referred to during discussion.

### Analysis

**Figure 1.**
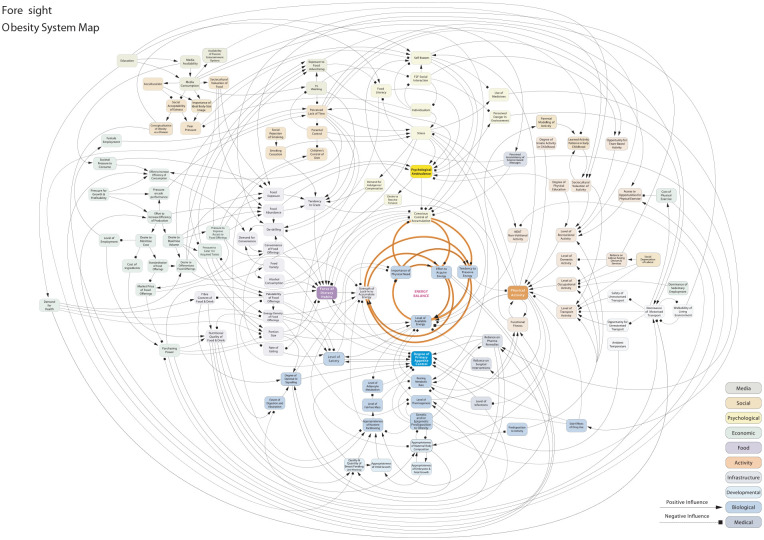
Foresight: Obesity system map. *Note.* Contains public sector information licensed under the Open Government License v3.0 (www.nationalarchives.gov.uk/doc/open-government-licence/version/3/).

Authors met immediately after the close of the workshop to review observation data and discussed early analytic memos. Analysis began with immersion in field notes from dialogue and interviews, and then expanded to include the wider dataset. We focused on identifying texts (such as policy papers, academic publications, images, etc.)—and interconnections between texts (e.g., by following the “journey” of the obesity map in Figure 1)—that were important in determining the conceptual content of complexity in applied health research, and how particular contributions related to efforts to specify complexity in particular ways. We worked iteratively from those texts, back to the original data, and to relevant theory to build a comprehensive understanding of the multiple meanings and uses of complexity.

Analysis was primarily informed by our theoretical interest in boundary-ordering devices which we discussed extensively in our data sessions (which are common in focused ethnography approaches, for example, see Knoblauch, 2005). We examined how boundary-ordering devices were formulated as dialogue about complexity unfolded at the workshop we studied. For each of these, we selected representative excerpts, drawing on extended sections of field notes and related texts for detailed analysis. We paid close analytic attention to points of tension in discussions of the nature and application of complexity in applied health research and its intersections with policy (e.g., disagreements in characterizing complexity or where complexity was assumed to be important but this was not operationalized). Throughout the analytic process, wider texts (e.g., abstracts) and contextual material (e.g., MRC framework) helped to set the scene. Authors’ participation in the workshop (including organizing) enabled a high degree of insider knowledge foundational to focused ethnographic work (Knoblauch, 2005). This also required a certain degree of reflexivity to balance our insider/outsider roles, foregrounding participant perspectives while being cognizant of how our own predispositions (disciplinary or otherwise) and the research process may have influenced knowledge production and interpretation. We discussed regularly (with other workshop participants or researchers without prior involvement in the workshop and early analysis), read widely, and worked reflexively to ensure we did not simply present a positive or largely descriptive assessment of the workshop.

## Findings

Participants debated complexity in multiple ways, which were illustrative of three boundary-ordering devices: (a) managing tensions between philosophical grounding of knowledge claims and need for practical application, (b) representing and abstracting complexity to open up communicative potential, and (c) articulating complexity as relational and accomplished. Each of these efforts to describe and use complexity served to continuously (re-)order boundaries across epistemic communities in particular ways, including with policy makers and practitioners as imagined audiences, thereby shaping what work on and with complexity was valued in applied health research.

### Managing Tensions Between Philosophical and Applied Meanings of Complexity

Workshop participants debated complexity both in terms of the need to articulate the concept in philosophically oriented ways and in relation to its practical application. There was a constant tension between these two perspectives: Some participants argued that philosophical issues must be worked out before any meaningful and coherent applications of complexity can proceed; others suggested that the articulation of epistemological and ontological assumptions does not have enough of an audience in applied health research (due to the research infrastructure not being positioned to support this, for example, as many funders in this area are not interested in theoretical questions of this nature).

In the example below, Participant 1 identified limitations in the articulation of complexity and suggested that a coherent standpoint on causality, ontology, and epistemology was essential to understanding how and why certain outcomes can be achieved. This comment triggered a discussion on whether a “philosophical” orientation is needed when thinking about complexity. Participant 2 conceded that complexity in itself would not be the key conceptual apparatus to approach intractable problems, but philosophical theories of causality are needed instead. In this short exchange, a number of philosophers were mentioned: Hume, Aristotle, and Kant. Participant 3 brought practical matters back into focus as representing a higher-order priority: Does it help if we have too many theories? Do we need more consensus and agreement to achieve “knowledge accumulation”? Guided by these different concerns, workshop discussions often relegated philosophical issues to the background, so that they could foreground practical application.

Participant 1: Thank you for an interesting series of talks, these have identified important issues. One issue is that we aren’t quite sure what we’re talking about; yes it’s some sort of complexity, but it seems to be interpreted differently by each speaker slightly. What might cause additional problems is that we don’t have a model of causation. Whatever we think complexity might be, or the outcome achieved as a result of complexity, etc., we need to start thinking about how these things are related to one another in any way.

Chair: You’re a realist!

Participant 1: [Laughs] It’s not important what position I come from, but it seems to be important to have some position on ontology. You introduced that [said to Chair]. That takes us on to epistemology. So if we don’t have some idea of what our knowledge claims are based on, that’s a problem. We need to have some sense of ontology. It makes the whole process not easy, but easier.

Chair [to panelists]: So should we be thinking a bit more philosophically?

Participant 2: Yes, I think we should. I wanted to title my talk “let’s not talk about complexity.” I think it’s a moot point whether obesity is a complex problem. Yes, we need to talk about causality. That’s obvious. Hume, Aristotle and Kant all addressed causality, they are my most favorite theories on the subject.

Participant 3: I was going to say we need a theory of complex systems. But what you’re saying is we have too many of these, we can’t settle in a place where we all know what it means and we agree, and can therefore build upon that the science that we need. So we need to settle, and then start building that evidence base.

The call to “think more philosophically” was met with enthusiasm by Participant 2 who contended that causality is at stake, rather than complexity. This remark points to how complexity is often treated as an undifferentiated, homogenized concept, whereas it encompasses a number of interlinked issues that need to be understood for their own merit (such as causality). At the same time that this comment invites philosophy to come closer to applied health research, it can be interpreted as transforming the problem into one that only philosophers are able (and potentially responsible) to tackle.

During the 2 days of the workshop, the discussion turned to ontological and philosophical foundations several times. At one point, the workshop chair invited two of the participants to discuss their (seemingly different) approaches to complexity, highlighting a common point of departure in that complex systems do not exist independently in the world, but gain meaning as part of experiential processes. Participant 4 referred to “philosophical pragmatism” and “teleological functionalism” to theoretically substantiate the tensions identified by the chair. In doing this, the discussion returned to the value of explicitly articulating philosophical assumptions about complex systems.

This time the value of a theoretical justification did not come at odds with practical applicability. Philosophical pragmatism became a device by which to bridge an orientation to philosophical explanation with more practical ways of addressing complexity in health care. Participant 5 linked pragmatism to specific aspects of the research infrastructure: “systematic reviews,” “randomized controlled trials,” and “best practices.” Without necessarily judging their utility, he foregrounded the currency in which a large part of applied health research trades. He argued for an urgent and pragmatic need to empirically look at what happens in the front line and establish a “different paradigm.” For this participant, it seemed that complexity-informed thinking held power in that it allowed everyday experiences of putting interventions into practice to be brought to the fore.

Chair: I want [Participant 4] and [Participant 5] to get into a dialogue. [Participant 4] it seemed to me is the only one so far to take a radical stance, saying that complexity is in the eye of the beholder. Many of us researchers go in assuming that complexity actually exists in the world and we are going to accurately describe it . . . this is an anthropologist saying we can construct a complex system and then study it. But it’s not out there in the world in the same way we think of it being. [Participant 5] is saying that complexity really is in the lived experience of people on the front line. But you’re both saying that stuff isn’t the “reality” we have to go and study; instead it is that which is salient to the individual at the experiential level.

Participant 4: I think this comes back to philosophical pragmatism. I have not talked about systems as things existing in the world at all. We tidy up the world as much as we can, this is teleological functionalism. The mess and experience of the world might relate to what [name] is saying—the patterns that people make to make sense of their world, and to cope within it.

Participant 5: Look we just want to do something with people on the front line. There are 11 systematic reviews and 75 RCTs (Randomized Controlled Trials) published every day. We need a different paradigm. We need to understand how people on the front lines actually deliver care before we do any more research on best practices.

In the above two excerpts, the discussion is underpinned by tensions in articulating philosophical foundations, while sustaining a concern for practical impact in the field of health research. The constant move between philosophy and practical application became a boundary-ordering device illustrating researchers’ efforts to order complexity in particular ways across their disciplinary boundaries (and imagined audiences). Our observations suggest that complexity cannot just be seen as either a philosophical or a practical problem, but instead gains currency as a concept advancing the importance of applied health research, because it allows for this constant foregrounding and backgrounding between the two perspectives, without excluding either as a focal point of interest. Throughout the workshop, these two seemingly contradictory views about what it means to work with complexity (philosophical explanation and practical application) *hung together* in ways that allowed participants to collectively discuss their different approaches in productive and useful ways.

### Representing and Abstracting Complexity

Workshop participants employed a second boundary-ordering device when discussing how to “represent” complexity using ready-made tools in the applied health research infrastructure. One tool used by researchers to portray complexity became central to discussions: the “causal loop diagram” (referred to elsewhere as a “foresight map,” “systems map,” or “spaghetti diagram”). Causal loop diagrams arose from the study of system dynamics and have been used to represent causal relationships between elements of a system since at least the 1950s (Richardson, 1986). A foresight map (Figure 1) that featured in more than one presentations was taken from a U.K. government report titled “Tackling Obesities: Future Choices” (Government Office for Science, 2007).

As workshop participants noted, causal loop diagrams are visually appealing. At first glance they appear to capture a wide range of interacting influences on a focal phenomenon. Embedded in the diagram are assumptions about the causal nature of relationships between influences and outcomes, depicted through lines and arrows. The foresight map in Figure 1 sparked lively discussion at the workshop about what exactly it represents, eliciting different opinions about its value. Some participants believed the foresight map to be overly abstract and simplistic, therefore missing the whole point of complexity, whereas others viewed it as a useful tool for communicating to non-academic stakeholders, transforming complexity into something tractable:

Participant 6: I always think with system maps, the problem with them is that they are too simple. It’s attempting to say it’s a complicated structure, but it’s completely unclear what the lines and arrows mean. That lack of precision renders the whole bit pretty much meaningless. These are systems maps about associations, not causation.

Chair: So it’s not at the level of precision you’re after?

Participant 7: That’s not the point of it. I think it’s deeply flawed, I accept the criticisms of the foresight map [the causal loop diagram shown in Figure 1]. It’s not there to allow you to reduce complex systems and eliminate causality. The primary purpose is to encourage policy people to approach a problem differently. I’m not saying you can model it precisely.

Participant 8: A map is better than nothing. It gives a sense of direction.

Participant 9: It’s a heuristic tool. I put whatever label on it I want and say I’m glad you can’t read this. It’s just to make the point that the system is complex. For a lot of audiences that is a novel concept.

The contributions presented here illustrate how the causal map has the potential to be employed flexibly, to accommodate varied conceptions of complexity and achieve different aims. In the first instance, the difference between association and causation comes to the fore as foundational in understanding complexity-informed research. “Precision” in representation is seen as important in advancing a complexity-informed understanding and *causality* returns to the surface as a central element in the discussion.

But the causal map is also assumed to be playing a different, primarily symbolic role. Participants suggested that in certain contexts of application, causality might not matter that much. The purpose of the causal loop diagram may not be to represent complexity in terms of causal influences, but to open up *communicative potential* across boundaries with different audiences (e.g., policy makers) when a common sense of direction needs to be developed. Representations of complexity act as a shortcut to developing the potential for collaborative work between stakeholders across boundaries characterized by different agendas and epistemological commitments.

Once again the conversation returned to practical import under a logic of applied utility through emphasis on communicative potential. This time the audiences for which complexity is mobilized were more clearly specified (i.e., policy makers). The final participant in the exchange suggested that these audiences need to be convinced that the “system is complex” in the first place—with an abstracted representation used to convince on complexity. Being able to communicate complexity seemed to encourage debates from multiple perspectives that could potentially lead to better ownership of challenges and common development of sustainable solutions. Effective communication across boundaries was also deemed important to legitimize and justify why certain problems are more difficult to tackle, in a way acceptable to all involved. However, given that complexity may not be amenable to “full” representation or to representation with “precision,” decisions may need to be made, implicitly or explicitly, about whose complexity counts.

### Complexity as Relational and Accomplished

The foresight map discussed in the previous section was intended to give “a sense of direction” according to one of the participants—but what direction one may ask? In reflecting on the workshop discussions, participants framed complexity not just as experiential but “always in relation to something else.” For instance, on Day 2, a participant highlighted that a complexity-informed understanding can only be generated by comparing and contrasting between different models, more or less simple:

Participant 9: From yesterday, I started to think: Is complexity always in relation to something else, a critique of someone else’s over-simplicity? [. . .] I was taken by the comment that these models, the spaghetti diagrams, themselves are oversimplifications. Any model is a simplification of reality—we are really trading simplifications about reality.

Use of the “trading simplifications” metaphor conveys the processes involved in building up an understanding of complexity in applied health research and highlights two things. First, that complexity should not be seen deterministically as a subjective attribute of an object or situation, but as constantly emerging, a precarious and practical accomplishment in the attempt of applied health researchers to make sense of the world. Our analysis highlighted that, for those participating in the workshop at least, this was the only way to make sense of complexity without pinning it down, that is, allowing the concept to work across boundaries and reconcile heterogeneous understandings. Second, the “trading” metaphor surfaced issues of power and exchange in representing complexity. The same participant went on to explain what she thought the research community should be asking:

Participant 9: Should we be focusing on the underlying motives for invoking complexity? Should we be looking for false beliefs? Who holds the power to leverage complexity as a way of thinking? These are the questions that most resonate with me.

In considering complexity as relational and accomplished, participants expressed that it becomes important to dig deeper in understanding why it is being invoked, to what ends, and toward what audiences. By asking who has the power to leverage complexity and in what ways, participants began to surface the interests at play in re-positioning boundaries around “good” applied health research. In this respect, the discussion on relationality became another boundary-ordering device; it pointed to the fluid nature of boundaries both around complexity and between groups, illustrating the dynamic nature of the ways in which complexity influences research practices in applied health research. From Star and Griesemer’s (1989) ecological perspective, this is not simply an issue of individual motives. Instead, individuals bring with them institutional influence that empowers them to make claims one way or the other. Their interests do not have symmetrical influence, and power plays a productive role in enabling the investment of greater resources into specific parts of the research enterprise.

## Discussion

Complexity-informed approaches have become popular in health research precisely because they signify a move away from linear ways of viewing the world and shift attention toward multiple, unpredictable, and interacting influences on long-standing problems. This focused ethnography has surfaced overlapping and divergent ways in which health researchers debate “good” complexity-informed research: one that manages the tension between being well-articulated philosophically and at the same time displaying direct application to practical problems, that uses different types of representations to communicate complexity without necessarily opening it up for scrutiny, and that sees complexity as relational and (often asymmetrically) accomplished for specific purposes and audiences. These three boundary-ordering devices characterized researchers’ discursive attempts to carve complexity in heterogeneous ways, while sustaining a broadly common sense of direction in the field of health research.

Recent literature is replete with debates on whether complexity-informed approaches should be better defined methodologically and operationalized more consistently. For example, a scoping review has argued that “conceptual confusion and inconsistent application hinders the operationalization of this potentially important perspective” (Thompson et al., 2016, p. 87). A bibliometric analysis has also pointed to the lack of practical guidance on methodological dimensions of complexity in ways that support researchers in operationalizing complexity theory (Churruca et al., 2019). Non-empirical articles have remained equally high-level, theoretically addressing features of complexity without expressing clear links to practical application (Churruca et al., 2019). Others, however, contest the extent to which complexity can be pinned down methodologically, recognizing that “any methodological choice emphasises some complexities and lets others fade into the background” (Broer et al., 2017, p. 135). In previous work, we have offered a series of principles (rather than prescriptive guidance) that could accompany a paradigm shift toward studying complexity; these included rich theorizing, generative learning, and pragmatic adaptation to changing contexts (Greenhalgh & Papoutsi, 2018).

Rather than seeking conceptual clarity or methodological uniformity in the accounts of our participants, in this current article, we examined how researchers hold together different meanings and purposes of complexity in interdisciplinary engagement. Acknowledging that attempts to neatly define or circumscribe complexity can be futile—either as an actor’s or as an analyst’s category (Broer et al., 2017)—Our interest is in highlighting ways in which complexity in applied health research can inspire a different understanding of how and why different voices become backgrounded in conventional research paradigms, and to support a commitment to following counter-arguments, working with contradictions, exploring tensions, and being critical about assumptions. Qualitative researchers can play a central role in advancing a reflexive approach to complexity-informed research, instead of highly formulaic applications. Qualitative researchers are also well placed to recognize where complexity is invoked in name only, rather than in substance to enhance (and where needed challenge) interdependencies and sense-making capacities. It became clear in the workshop that a complexity-informed approach requires interdisciplinary commitment, for example, to be able to unpack questions around causality and the nature of knowledge claims. Most of all, this interdisciplinary commitment becomes necessary to engage in a “turn to complexity” which would firmly place reflexive qualitative approaches at the center of the research infrastructure, when applied health research currently trades to a large extent in formulaic systematic reviews and experimental designs.

In their social study of environmental scientists, Shackley and Wynne (1996) outlined what they suggest as the dual accomplishments of boundary-ordering devices: to “facilitate interaction, translation, and cooperation between science and policy worlds while still ordering the relations between science and policy so as to sustain the special cultural authority of science” (p. 280). Similarly, in our case, health researchers centered on policy makers and practitioners as imagined audiences and framed complexity-informed approaches as responsive to their needs. For qualitative health researchers, the question then becomes, how do we communicate and translate complexity for different audiences without reverting back to the linear to make our messages more easily transferable? There are no easy answers, but as one illustrative example, Lanham et al. (2013) employ case studies to highlight the importance of harnessing relationships and self-organization, remaining alert to local needs for modifications, and supporting sense-making, in the scale-up and spread of health care interventions. As another example, more recently, the MRC has commissioned our team to develop guidance on the use of case study methods to appreciate the role of context in shaping complex interventions (Paparini et al., in press).

Although our analysis is primarily based on a 2-day, high-profile workshop, the range of extensive discussions and sophisticated contributions from leading academics has generated a rich dataset reflecting some of the key debates in the field. Despite the workshop attracting international speakers, there was regrettably little representation from low- and middle-income countries which may have contributed different perspectives to the role of complexity in health research. By using boundary-ordering devices as lens for our analysis, we have been able to demonstrate both the continued relevance of complexity as a theoretical approach for the development of scientific fields and have opened up a more nuanced understanding of the tensions through which complexity develops as a conceptual lens in health research. Although complexity may only be a concept, its rhetorical power acts as a vehicle for positioning the value of health research and its ability to address “intractable” problems.

## Conclusion

Through this focused ethnography, we have surfaced some of the ways in which epistemic communities hold together heterogeneous meanings and inherent tensions around what counts as good complexity-informed health research. This serves a dual purpose in not only sustaining a broad sense of common direction (despite heterogeneous meanings) within this interdisciplinary field but also exhibiting scientific and applied value for policy and practice. Qualitative health researchers could strengthen dialogue with conventional research paradigms through a more reflexive approach to complexity-informed research that treads the balance between philosophical articulation and practical relevance, and fosters an understanding of interdependencies, tensions, contradictions, and emergence.
